# Associations of Major Dietary Patterns With Breast Cancer Among Iranian Women: A Hospital‐Based Case–Control Study

**DOI:** 10.1002/cnr2.70341

**Published:** 2025-09-09

**Authors:** Hamidreza Razmi, Mohammad Hadi Nikbakht, Paria Jemsi, Sima Jafarirad, Maedeh Raeisi Zadeh, Samira Razzaghi, Ali Veissi, Farnush Bakhshimoghaddam

**Affiliations:** ^1^ Student Research Committee Ahvaz Jundishapur University of Medical Sciences Ahvaz Iran; ^2^ Nutrition and Metabolic Diseases Research Center, Clinical Sciences Research Institute Ahvaz Jundishapur University of Medical Sciences Ahvaz Iran; ^3^ Department of Nutrition, School of Allied Medical Sciences Ahvaz Jundishapur University of Medical Sciences Ahvaz Iran; ^4^ Department of Biostatistics and Epidemiology School of Public Health, Ahvaz Jundishapur University of Medical Sciences Ahvaz Iran; ^5^ Department of Clinical Oncology Golestan Hospital, Ahvaz Jundishapur University of Medical Sciences Ahvaz Iran

**Keywords:** breast cancer, dietary patterns, factor analysis, Western diet

## Abstract

**Background and Aims:**

Breast cancer is the most commonly diagnosed cancer in women worldwide. Several studies have investigated the relationship between breast cancer and specific foods or nutrients, rather than examining an overall dietary pattern. This study aims to investigate the association between breast cancer and the predominant dietary pattern in Ahvaz city.

**Methods:**

This hospital‐based case–control study was conducted on 106 women with breast cancer and 107 controls. Dietary intake data were collected using a 147‐item food frequency questionnaire. We merged the data on the 147 foods into 20 food groups to identify major dietary patterns. Factor analysis with varimax rotation was then employed to determine the primary dietary patterns. Binary logistic regression was used to assess the association between dietary patterns and breast cancer, with adjustment for potential confounders.

**Results:**

Three dietary patterns were identified: healthy, western, and traditional. The Western dietary pattern was robustly associated with a higher risk of breast cancer in premenopausal women (OR = 4.22; 95% CI: 1.09, 16.31; *p* = 0.03) in the adjusted model. However, no association was found between a healthy and traditional pattern and breast cancer.

**Conclusion:**

These findings suggest that the Western dietary pattern is positively associated with breast cancer risk in premenopausal Iranian women.

**Abbreviations**
BMI: body mass index
FFQ: food frequency questionnaire
MET: metabolic equivalent minutes
SSB: Sugar‐strengthened beverage
WHR: waist‐hip ratio

## 1 Introduction

Breast cancer is the primary cancer in females in developed countries, and there has been an increase in incidence and mortality in low‐ and middle‐income societies [1]. The World Health Organization predicts that up to about 2.3 million women will be diagnosed with breast cancer by 2050 [2]. Breast cancer is the sixth leading cause of death in Iran, accounting for 12.5% of all cancers [3]. Several risk factors for breast cancer are both proven and controversial. There is some evidence that breast cancer risk factors include aging, marital status (single), socioeconomic status (upper‐middle income), history of breast or other female malignancy, fertility and hormonal issues, unhealthy lifestyle (obesity, smoking, alcohol drinking, and physical inactivity), and nutritional transition [4].

Due to socioeconomic conditions, geographical differences, and cultural variations in food habits, preferences, and availability, dietary patterns are expected to be diverse among different populations [5]. Up to now, most epidemiological studies investigating the potential role of dietary habits in developing breast cancer have tended to focus on Western countries [6, 7]. However, several studies conducted in Asian populations have suggested that, in recent years, people have shifted from healthy dietary patterns to Western dietary patterns characterized by a reduced consumption of vegetables, fruits, whole grains, and legumes, and an increased consumption of red and processed red meat, fast food, and refined grains. This may influence the breast cancer risk among Asian women [8].

While numerous studies have investigated dietary patterns and breast cancer risk, most evidence derives from Western or East Asian populations [6, 7, 8], with limited data from multiethnic regions of Southwest Asia, particularly Iran. Existing studies in Iran have reported inconsistent associations [9, 10, 11], likely due to regional dietary heterogeneity, cultural diversity, and varying methodologies. In two case–control studies, adherence to a healthy dietary pattern characterized by a high intake of vegetables, fruits, legumes, nuts, low‐fat dairy products, and plant‐based oils (e.g., olive oil) was associated with a reduced risk of breast cancer. In contrast, adherence to an unhealthy dietary pattern emphasized refined grains, red/processed meats, sugar‐sweetened beverages (SSBs), fast food, and sweets was associated with a significantly increased risk of breast cancer among Iranian women [9, 10]. In another study, however, there has been no association between a healthy dietary pattern and the risk of developing breast cancer [11].

This study addresses critical gaps by focusing on Ahvaz, an industrial, multiethnic city with unique dietary habits shaped by Arab, Lur, and Persian influences, offering insights into underrepresented populations. Our study population's diversity allowed the first examination of how breast cancer risks vary across ethnic dietary preferences within Iran. Therefore, this hospital‐based case–control study was designed to investigate the associations between breast cancer and the predominant dietary patterns in Ahvaz, particularly the Traditional pattern (high‐fat dairy, solid oils, and salty foods) and Westernized patterns emerging in urban settings.

## 2 Materials and Methods

**FIGURE 1:**
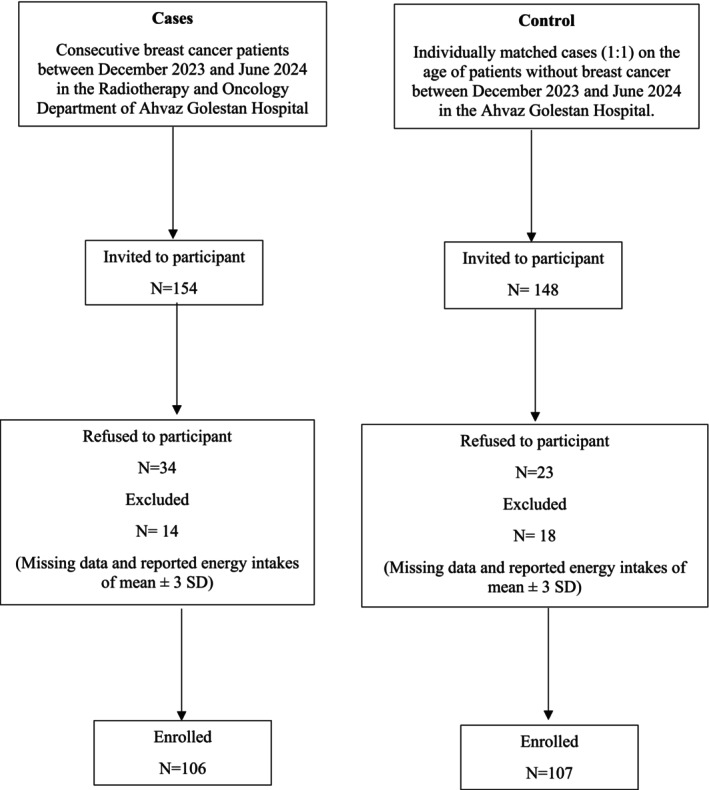
Flowchart of enrolment of cases and controls.

We conducted a hospital‐based case–control study among Iranian women aged 18 years and above who were admitted to Golestan Hospital, a referral hospital in Ahvaz, between December 2023 and June 2024. The case group consisted of women with a histopathological confirmed diagnosis of breast cancer who were referred to the Radiotherapy and Oncology Department of Ahvaz Golestan Hospital. The control group comprised patients admitted to other departments of Golestan Hospital for various non‐oncological conditions that are not known to alter long‐term dietary patterns (e.g., diabetes, gastrointestinal disorders, and chronic renal disease), smoking, or alcohol dependence. Medical disorders in the controls included acute surgical conditions, orthopedic and trauma disorders, and otolaryngological disease with ≤ 3‐day hospital stays. All controls had a stable body weight (±3 kg) in the preceding 6 months and had not made significant dietary changes in the past year. Cases and control groups were frequency‐matched based on age (±5 years). On average, questionnaires were administered 1.6 ± 2.7 years after breast cancer diagnosis (range: 0.5–5 years). In the meantime, a total of 148 control subjects were invited, and 125 participated in the study, resulting in a response rate of 84.5%. However, a total of 106 cases and 107 controls remained in the final analysis after excluding 18 controls and 14 cases with missing data and reported energy intakes (mean ±3 SD) (Figure 1).

### 2.1 Demographic Data

Structured questionnaires were used to collect demographic data, lifestyle characteristics (including physical activity and smoking), socioeconomic status, hormonal and fertility status, and family history of breast and other cancers during face‐to‐face interviews conducted by trained professionals.

### 2.2 Anthropometric Assessment and Physical Activity

With the participants wearing minimal clothing and no shoes, body weight was measured to the nearest 100 g using digital scales. The height was measured to the nearest 0.1 cm in the standing position with no shoes on. Weight (kg) was divided by height (m^2^) to calculate body mass index (BMI) (kg/m^2^). Waist and hip circumferences were recorded to the nearest 0.1 cm using a flexible metric tape measure with participants standing. Waist circumference was measured around the abdomen at the level of the umbilicus. Hip circumference was measured at the point of maximum posterior buttock extension in a horizontal plane. The waist‐to‐hip ratio (WHR) was calculated as the waist circumference (cm) divided by the hip circumference (cm).

Physical activity was evaluated using the short version of the International Physical Activity Questionnaire (IPAQ) and reported as metabolic equivalent minutes per week (MET/min/week), which has been previously validated in Iranian adults [12].

### 2.3 Dietary Assessment

A semi‐quantitative food frequency questionnaire (FFQ) designed explicitly for the Iranian population was used to assess the dietary intake of the study participants. This FFQ included 147 food items and had been shown in previous research to be a reliable and valid measure of Iranian dietary habits [13, 14]. Participants were asked about the frequency of consumption of standard portion sizes over the past year. Participants reported the frequency with which they consumed the food items in the FFQ, on a daily, weekly, monthly, and yearly basis. All of the information was converted into values for the daily intake. Household measures were used to estimate the grams of portion sizes consumed daily [15] using Nutritionist IV software, total daily energy, protein, carbohydrates, fat, and fiber intakes were calculated for each participant based on the grams of food items on the questionnaire.

### 2.4 Statistical Analysis

To determine the main dietary patterns, the data on the 147 food items were first grouped into 20 food groups based on the similarity of their nutrient content. Food group intakes were energy‐adjusted by the residual method [16]. We employed factor analysis with varimax rotation to identify the primary dietary patterns. Factor retention was determined through: (1) scree plot inflection, (2) eigenvalues > 1.4 (conservative vs. conventional > 1 cutoff), (3) parallel analysis, and (4) interpretability. We identified three interpretable dietary patterns, labeled based on the loadings of the items in each factor and previous literature. The three‐factor solution was selected as it optimally balanced explained variance (32.89%) against clinical utility and stability. Dietary pattern scores were calculated, and each participant received a score for each identified pattern by summing the weighted intakes of food items, based on their factor loadings. We classified the participants according to the quartiles of their dietary pattern scores.

Mean (SD) and frequency (percentage) were used for continuous and categorical variables, respectively. Kolmogorov–Smirnov and *Q*–*Q* plots were used to assess the normality of constant data. We used the independent samples Student's *t*‐test and *χ* ^2^ test, where appropriate, for comparing study participant characteristics. Variables were compared across quartiles of dietary patterns using a one‐way analysis of variance and *χ* ^2^ tests. The odds ratios (ORs) and their 95% confidence intervals (CIs) for breast cancer risk by quartiles of each dietary pattern score were calculated using multivariate logistic regression in crude or adjusted models. Associations were adjusted for age, BMI, WHR, energy intake, smoking status, physical activity, education, employment status, duration of diagnosis, treatment modalities (chemotherapy, radiotherapy, surgery, and hormonal therapy), and family history of breast or other cancer. To visualize the association between dietary patterns and breast cancer risk, we generated a forest plot using GraphPad Prism 8 (GraphPad Software, San Diego, CA). The plot displays adjusted ORs and 95% CIs for the highest versus lowest quartile (Q4 vs. Q1) of each dietary pattern, stratified by menopausal status. All analyses were conducted using SPSS software (version 18). *p*‐values < 0.05 were considered significant.

## 3 Result

Table 1 shows the descriptive characteristics of the participants by case–control status. Compared to controls, cases were more likely to have a higher WHR (*p* < 0.001), higher energy intake (*p* = 0.04), and lower dietary fiber intake (*p* < 0.001). Also, compared to the control group, the case group tended to be more illiterate and less educated (*p* = 0.04), more likely to be post‐menopausal (*p* < 0.001), less employed (*p* = 0.002), have a higher percentage of a miscarriage history (*p* = 0.05), more family history of breast (*p* = 0.003) or other cancers (*p* < 0.001).

**TABLE 1:** Descriptive characteristics of the participants by case/control status.

	Case	Control	*p* ^a^
Age	49.32 ± 9.10	48.84 ± 8.51	0.69
Weight	71.33 ± 15.02	70.67 ± 14.79	0.75
BMI	27.66 ± 5.38	27.49 ± 6.06	0.83
WHR	0.89 ± 0.07	0.83 ± 0.11	**< 0.001**
Education (*n*, %)			**0.04**
Illiterate	10 (9.4%)	5 (4.7%)	
Low	51 (48.1%)	39 (36.4%)	
Medium	22 (20.8%)	22 (20.6%)	
High	23 (21.7%)	41 (38.3%)	
Menopausal (*n*, %)			**< 0.001**
Yes	44 (41.5%)	14 (13.1%)	
No	62 (58.5%)	93 (86.9%)	
Marital status (*n*, %)			0.32
Single	17 (16.2%)	23 (21.5%)	
Married	88 (83.8%)	84 (78.5%)	
Employment status (*n*, %)			**0.002**
Housekeeper	93 (87.7%)	75 (70.1%)	
Employee	13 (12.3%)	32 (29.9%)	
Birth control pills (*n*, %)			0.07
Yes	40 (37.7%)	53 (50.0%)	
No	66 (62.3%)	53 (50.0%)	
Number of children (*n*, %)			0.24
< 5	87 (82.1%)	94 (87.9%)	
≥ 5	19 (17.9%)	13 (12.1%)	
Miscarriage history (*n*, %)			0.05
Yes	24 (24.7%)	15 (14.0%)	
No	73 (75.3%)	92 (86.0%)	
Family history of breast cancer (*n*, %)			**0.003**
Yes	27 (26.0%)	10 (10.0%)	
No	77 (74.0%)	90 (90%)	
Treatment modalities (*n*, %)			
Chemotherapy	49 (46.2%)	—	
Radiotherapy	37 (34.9%)	—	
Surgery	16 (15.1%)	—	
Hormonal therapy	4 (3.8%)	—	
Duration of diagnosis (year)	1.6 ± 2.7	—	
Family history of another cancer (*n*, %)			**< 0.001**
Yes	46 (44.2%)	7 (7.4%)	
No	58 (55.8%)	87 (92.6%)	
Current smoker (*n*, %)			0.10
Yes	8 (7.6%)	3 (2.8%)	
No	97 (92.4%)	104 (97.2%)	
Physical activity (MET/min/week)	37.24 ± 6.22	37.26 ± 5.96	0.97
Energy intake	2769.64 ± 964.46	2502.52 ± 932.40	**0.04**
Protein intake	97.97 ± 32.69	89.90 ± 38.97	0.10
Carbohydrate intake	445.48 ± 197.06	397.79 ± 157.56	0.05
Fat intake	72.08 ± 27.48	67.06 ± 30.71	0.21
Dietary fiber intake	27.06 ± 11.71	48.80 ± 19.85	**< 0.001**

*Note:* Data are presented as mean ± SD or percent (*n*). Statistically significant values (*p*‐value < 0.05) are highlighted in bold.

Abbreviations: BMI, body mass index; WHR, waist‐hip ratio.

^a^ Obtained from the independent‐sample *t*‐test, chi‐squared, or Fisher test, where appropriate.

Three dietary patterns were identified by principal components analysis. Table 2 presents the factor scores of the three dietary patterns, along with the names assigned to each pattern. The “healthy dietary pattern” was heavily weighted towards vegetables, dried fruit, legumes, nuts, liquid oil, low‐fat dairy products, olives, and olive oil. The “Western dietary pattern” consisted mainly of more refined grains, red and organ meats, SSBs, sweets and desserts, and fast food. The “traditional dietary pattern” was characterized by a variety of salty foods, tea and coffee, high‐fat dairy products, snacks, white meat (poultry, fish, and fish products), eggs, solid oils, and whole grains. The three factors explained 32.89% of the variance in dietary patterns, with healthy, Western, and traditional patterns accounting for 13.41%, 10.26%, and 9.22%, respectively. Table 3 outlines the foods and range of approximate daily servings for the three identified dietary patterns among Iranian women.

**TABLE 2:** Factor loading matrix for the major dietary patterns identified among the sample of Iranian women.

	Healthy dietary pattern	Western dietary pattern	Traditional dietary pattern
Vegetable	0.835		
Dried fruit	0.667		
Fruit	0.486		
Legume and nut	0.421		
Liquid oil	0.405		
Low‐fat dairy	0.395		
Olive and olive oil	0.286		
Refined grain		0.754	
Red and organ meat		0.654	
SSB		0.548	
Sweets and desserts		0.361	
Fast food		0.254	
Salty			0.624
Tea and coffee			0.524
High‐fat dairy			0.459
Snack			0.427
White meat^a^			0.363
Egg			0.358
Solid oil			0.327
Whole grain			0.111
Variance explained (%)	13.41	10.26	9.22

Abbreviation: SSB, sugar‐strengthened beverage.

^a^ Included poultry, fish, and fish products.

**TABLE 3:** Foods and approximate servings in three dietary patterns.

Dietary pattern	Foods	Approximate daily servings (mean ± SD)
Healthy dietary pattern	Vegetables	2–3 servings/day
	Dried fruit	1–2 servings/day
	Fruit	2–3 servings/day
	Legumes and nuts	1–2 servings/day
	Liquid oil (e.g., olive)	1–2 servings/day
	Low‐fat dairy	1–2 servings/day
	Olive and olive oil	1 serving/day
Western dietary pattern	Refined grains	3–4 servings/day
	Red and organ meat	2–3 servings/week
	Sugar‐sweetened beverages	1–2 servings/day
	Sweets and desserts	3–4 servings/week
	Fast food	1–2 servings/week
Traditional dietary pattern	Salty foods	2–3 servings/day
	Tea and coffee	3–4 servings/day
	High‐fat dairy	1–2 servings/day
	Snacks	1–2 servings/day
	White meat (poultry/fish)	2–3 servings/week
	Eggs	1–2 servings/week
	Solid oil (e.g., butter)	1 serving/day
	Whole grains	1–2 servings/day

Compared to the lowest quartile (Q1) of the healthy pattern, the highest quartile (Q4) had a lower mean BMI, a higher mean total carbohydrate intake, a higher mean vegetable intake, a higher percentage of highly educated individuals, and a higher number of employees. The Q4 traditional diet group consumed a greater variety of foods, including more eggs, tea, coffee, and salty foods. In the Western dietary pattern, the mean BMI, consumption of refined grains, red and processed meats, and SSBs were higher in the fourth quarter (Q4) compared to the other quarters. Compared to Q1 for the healthy and western dietary pattern, Q4 had a high mean for fruit, dried fruit, liquid oil, and olive and olive oil. The Q4 healthy and traditional dietary pattern group had the highest mean intake of dietary fiber, whole grains, high‐ and low‐fat dairy products, and white meat (including poultry, fish, and fish products) compared to the Q1 group. In three dietary patterns, compared to Q1, Q4 had a higher mean WHR, as well as higher consumption of fast food, sweets and desserts, legumes and nuts, and a higher percentage of married women (Table 4).

**TABLE 4:** Sample characteristics for the lowest (Q1) and highest (Q4) quartiles of three dietary patterns (*N* = 213).

	Healthy dietary pattern score		Western dietary pattern score		Traditional dietary pattern scores	
	Q1	Q4	Q1	Q4	Q1	Q4
Age	46.15 ± 9.92	48.90 ± 8.29	49.54 ± 8.64	46.77 ± 8.80*	48.45 ± 9.71	45.67 ± 9.38
Weight	72.68 ± 15.35	69.99 ± 15.79	71.44 ± 15.55	72.33 ± 15.57	70.89 ± 16.10	71.16 ± 14.71
BMI	27.65 ± 6.19	26.14 ± 4.97*	27.07 ± 5.07	28.81 ± 5.95	26.82 ± 5.04	27.51 ± 5.18
WHR	0.89 ± 0.12	0.83 ± 0.08*	0.84 ± 0.08	0.89 ± 0.8*	0.83 ± 0.08	0.88 ± 0.09*
Education (*n*, %)						
Illiterate	3 (4.2%)	8 (11.3%)	7 (9.9%)	4 (5.6%)	7 (9.9%)	7 (9.9%)
Low	36 (50.7%)	33 (46.5%)	27 (38.0%)	33 (46.5%)	29 (40.8%)	27 (38.0%)
Medium	16 (22.5%)	5 (7.0%)	15 (21.1%)	13 (18.3%)	15 (21.1%)	14 (19.7%)
High	16 (22.5%)	25 (35.2%)	22 (31.0%)	21 (29.6%)	20 (28.2%)	23 (32.4%)
Marital status (*n*, %)						
Single	17 (23.9%)	9 (12.9%)	13 (18.3%)	7 (10.0%)	17 (23.9%)	8 (11.4%)
Married	54 (76.1%)	61 (87.1%)	58 (81.7%)	63 (90.0%)	54 (76.1%)	62 (88.6%)
Employment status (*n*, %)						
Housekeeper	58 (81.7%)	53 (74.6%)	58 (81.7%)	55 (77.5%)	55 (77.5%)	55 (77.5%)
Employee	13 (18.3%)	18 (25.4%)	13 (18.3%)	16 (22.5%)	16 (22.5%)	16 (22.5%)
Smoking history						
Yes	4 (5.6%)	5 (7.1%)	4 (5.6%)	5 (7.1%)	1 (1.4%)	6 (8.6%)
No	67 (94.4%)	65 (92.9%)	67 (94.4%)	65 (92.9%)	70 (89.6%)	64 (91.4%)
Physical activity (MET)^#^	36.49 ± 5.46	38.12 ± 7.25	37.28 ± 5.06	38.59 ± 6.18	36.98 ± 5.15	37.26 ± 7.29
Energy intake	2858.70 ± 961.15	2500.19 ± 912.79	2567.93 ± 866.39	2627.26 ± 1030.23	2591.45 ± 954.92	2650.88 ± 856.69
Carbohydrate	474.47 ± 175.60	386.19 ± 174.46*	409.61 ± 171.46	427.54 ± 184.93	418.66 ± 155.14	424.23 ± 189.22
Protein	100.34 ± 35.14	91.64 ± 39.01	93.13 ± 39.27	90.13 ± 39.27	92.89 ± 31.63	93.50 ± 33.62
Fat	69.53 ± 28.74	67.65 ± 30.26	65.18 ± 31.74	70.70 ± 30.23	69.00 ± 29.62	72.35 ± 29.93
Dietary fiber	33.80 ± 23.69	46.32 ± 15.56*	39.61 ± 15.62	35.18 ± 22.76	33.30 ± 19.31	39.07 ± 16.53*
Whole grain	454.81 ± 166.98	251.14 ± 172.56*	333.37 ± 192.71	354.04 ± 150.74	303.64 ± 190.68	376.43 ± 149.64*
Refined grain	14.99 ± 22.82	28.45 ± 61.01	6.47 ± 9.44	61.20 ± 88.11*	32.25 ± 71.18	21.13 ± 33.61
Fruit	267.04 ± 151.34	691.26 ± 403.85*	353.71 ± 237.22	544.36 ± 423.24*	432.21 ± 402.73	414.36 ± 257.72
Vegetable	236.49 ± 100.88	682.38 ± 381.72*	460.55 ± 388.40	401.11 ± 277.83	368.01 ± 313.81	442.96 ± 313.34
Dried fruit	11.56 ± 13.63	70.82 ± 67.20*	50.18 ± 65.80	30.88 ± 35.12*	28.87 ± 47.31	38.36 ± 49.26
High‐fat dairy	124.43 ± 101.92	175.29 ± 128.01*	146.89 ± 106.21	155.04 ± 148.09	91.54 ± 66.05	209.74 ± 138.77*
Low‐fat dairy	50.72 ± 61.71	164.91 ± 138.57*	66.93 ± 83.84	184.85 ± 162.72*	162.40 ± 157.99	76.96 ± 80.30*
White meat	60.31 ± 31.91	87.14 ± 37.71*	70.55 ± 35.75	72.05 ± 34.27	55.29 ± 28.14	85.96 ± 37.09*
Red and organ meat	15.50 ± 12.91	13.84 ± 15.76	8.91 ± 6.57	27.23 ± 22.53*	15.80 ± 15.45	17.15 ± 16.83
Solid oil	5.79 ± 9.11	9.71 ± 15.38	3.71 ± 6.75	12.35 ± 15.55*	5.96 ± 8.74	13.14 ± 16.84*
Liquid oil	6.15 ± 4.47	11.16 ± 9.14*	9.93 ± 8.47	6.84 ± 6.32*	6.71 ± 5.00	8.49 ± 7.51
Egg	19.37 ± 19.14	29.81 ± 27.96	26.24 ± 29.63	23.46 ± 27.25	15.11 ± 14.05	35.32 ± 35.48*
Fast food	23.17 ± 25.02	8.10 ± 16.97*	6.12 ± 9.63	24.58 ± 29.28*	11.22 ± 18.92	23.86 ± 27.88*
Snack	9.5 ± 12.01	2.31 ± 5.13*	2.95 ± 4.02	9.26 ± 12.86*	3.56 ± 6.13	10.37 ± 12.36*
Sweets and desserts	32.64 ± 26.06	31.28 ± 23.32	23.70 ± 17.54	43.18 ± 31.81*	25.28 ± 20.00	47.83 ± 28.39*
SSB	69.82 ± 70.90	82.88 ± 70.91	28.56 ± 33.72	143.21 ± 203.81*	57.92 ± 93.15	76.28 ± 85.01
Tea and coffee	648.96 ± 713.61	504.43 ± 370.77	528.72 ± 455.91	592.18 ± 655.45	294.05 ± 271.78	862.57 ± 697.64*
Legume and Nut	34.18 ± 22.04	84.14 ± 37.14*	51.82 ± 37.37	77.58 ± 45.08*	54.40 ± 39.04	76.22 ± 46.41*
Olive and olive oil	0.55 ± 0.99	1.81 ± 3.54*	0.74 ± 2.82	1.75 ± 2.59*	0.87 ± 1.73	1.09 ± 2.04
Salty	34.69 ± 42.42	31.79 ± 49.24	28.77 ± 46.68	32.58 ± 49.61	10.61 ± 15.46	60.65 ± 67.93*

Abbreviations: BMI, body mass index; SSB, sugar‐strengthened beverage; WHR, waist‐hip ratio.

^*^ A significant difference (*p* < 0.05) between the highest (Q4) and lowest (Q1) quartiles for that variable, assessed by an independent‐samples *t*‐test.

^#^ Physical activity based on metabolic equivalent minutes per week.

**FIGURE 2:**
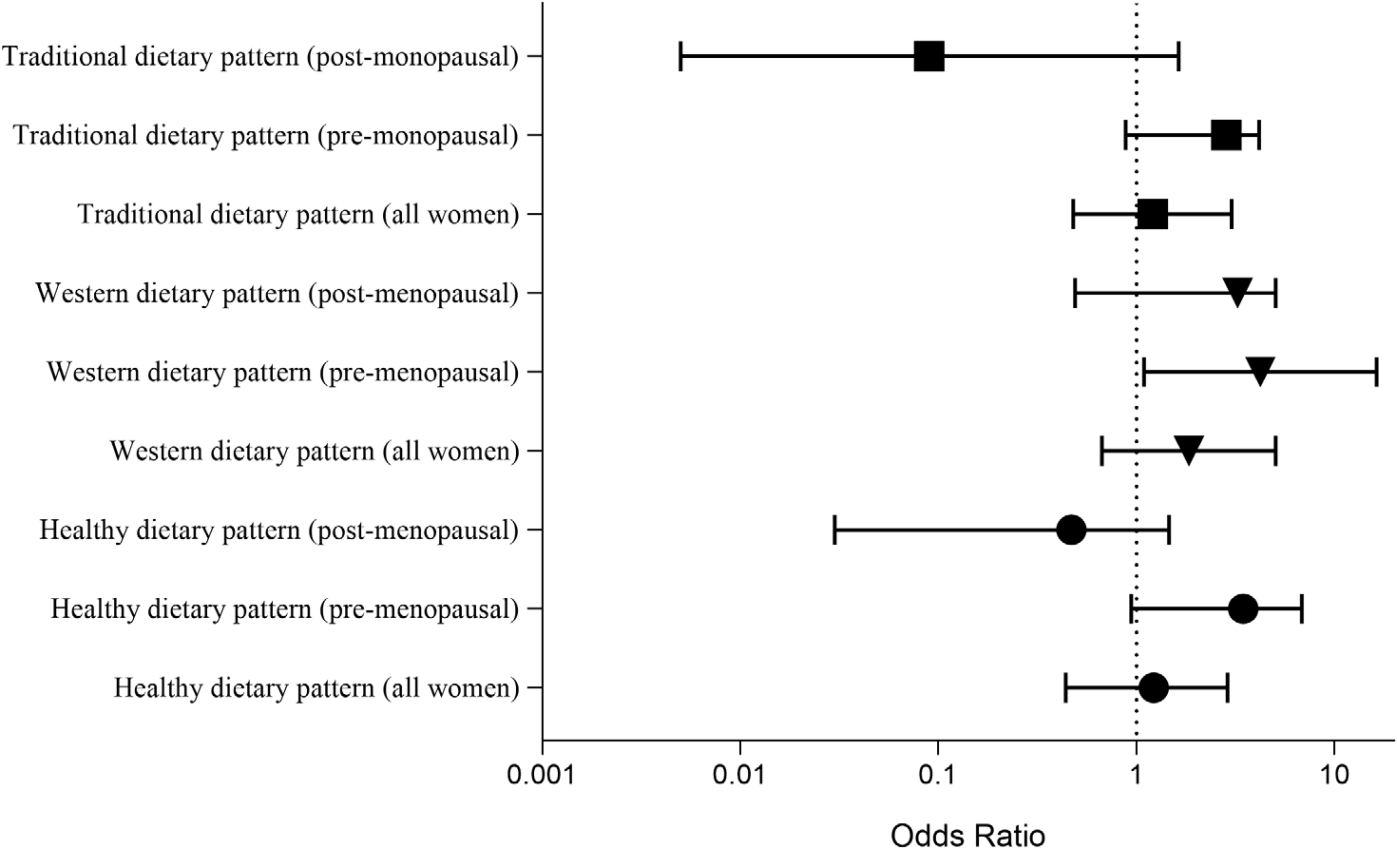
Forest plot of breast cancer risk associated with the highest adherence (Q4) to dietary patterns compared to the lowest adherence (Q1). Results are stratified by menopausal status and shown for all women combined. All models used the most adjusted covariates. The dashed vertical line indicates no effect (OR = 1).

In both the crude and adjusted models, no association was found between the three dietary patterns and breast cancer risk among women. However, after stratification by menopausal status, we found a positive and significant association between Western dietary patterns and breast cancer risk in premenopausal women in the adjusted model (OR = 4.22; 95% CI: 1.09, 16.31; *p* = 0.03) (Table 5 and Figure 2).

**TABLE 5:** The odds ratio (OR) and 95% confidence interval (95% CI) of breast cancer across quartiles of three dietary patterns^a^.

Dietary pattern	Quartile of dietary pattern score				*p*‐trend
	Q1	Q2	Q3	Q4	
Healthy dietary pattern score					
All women					
Crude	1	0.63 (0.29–1.36)	0.57 (0.26–1.22)	0.93 (0.43–1.99)	0.78
Adjusted	1	0.53 (0.20–1.39)	0.53 (0.21–1.37)	1.12 (0.44–2.88)	0.80
Pre‐menopausal					
Crude	1	0.87 (0.33–2.31)	1.12 (0.43–2.89)	1.67 (0.66–4.22)	0.90
Adjusted	1	0.82 (0.23–2.89)	1.56 (0.42–5.75)	3.46 (0.94–6.84)	0.07
Post‐menopausal					
Crude	1	0.40 (0.05–2.83)	0.13 (0.02–0.80)	0.44 (0.05–3.74)	0.38
Adjusted	1	0.39 (0.02–1.67)	0.10 (0.007–1.56)	0.47 (0.03–1.46)	0.29
Western dietary pattern score					
All women					
Crude	1	0.81 (0.39–1.69)	1.42 (0.68–2.97)	1.34 (0.60–2.99)	0.24
Adjusted	1	0.44 (0.17–1.16)	1.21 (0.49–2.94)	1.84 (0.67–5.04)	0.08
Pre‐menopausal					
Crude	1	1.86 (0.74–4.68)	2.34 (0.92–6.01)	2.16 (0.75–6.17)	0.11
Adjusted	1	1.52 (0.45–5.20)	2.01 (0.66–6.11)	4.22 (1.09–16.31)	**0.03**
Post‐menopausal					
Crude	1	0.17 (0.03–1.12)	1.06 (0.15–7.34)	0.48 (0.09–2.60)	0.69
Adjusted	1	1.87 (0.69–5.05)	2.97 (0.96–5.19)	3.24 (0.49–5.03)	0.53
Traditional dietary pattern score					
All women					
Crude	1	0.92 (0.43–1.99)	0.57 (0.26–1.22)	0.86 (0.40–1.84)	0.44
Adjusted	1	0.79 (0.31–2.01)	1.10 (0.45–2.70)	1.21 (0.48–3.02)	0.89
Pre‐menopausal					
Crude	1	0.87 (0.35–2.17)	1.21 (0.47–3.13)	1.52 (0.59–3.88)	0.54
Adjusted	1	1.32 (0.42–4.11)	1.23 (0.40–3.79)	2.84 (0.88–4.16)	0.16
Post‐menopausal					
Crude	1	0.41 (0.06–2.58)	0.75 (0.05–2.87)	0.19 (0.03–1.13)	0.08
Adjusted	1	0.13 (0.005–3.72)	0.44 (0.004–4.84)	0.09 (0.005–1.63)	0.06

*Note:* Statistically significant values (*p*‐value < 0.05) are highlighted in bold.

^a^ Adjusted for age, BMI, WHR, energy intake, smoking status, physical activity, education, employment status, treatment modalities, duration of diagnosis, and family history of breast or other cancer.

## 4 Discussion

In the current hospital‐based case–control study, we identified three main dietary patterns: healthy, Western, and traditional. The study's results showed that adherence to a Western dietary pattern increased the risk of breast cancer in premenopausal women. However, there was no statistically significant association between other dietary patterns and breast cancer risk in both crude and adjusted models.

Prior research conducted in Iran focused on “healthy” vs. “unhealthy” dichotomies [9, 11]. Ahvaz's dietary patterns (e.g., high consumption of traditional salty foods, liquid oils, and organ meats) differ markedly from those in Tehran or Northern Iran, where prior studies were conducted [9, 11]. We uniquely identified a traditional dietary pattern (characterized by salty foods, solid oils, and high‐fat dairy) prevalent in Southwest Iran, providing data on culturally relevant foods that are often overlooked elsewhere. There is evidence regarding the association of specific dietary patterns and breast cancer throughout the world, such as Western patterns, Eastern patterns, and Mediterranean patterns [17]. The Western dietary pattern is characterized by high intakes of energy, animal protein, saturated fat, and sugar, and is represented by developed countries in Europe and North America [18]. The Eastern pattern is more vegetarian and less animal‐based, typical of Asian countries [19]. The Mediterranean diet, typical of the Mediterranean area, is rich in fish, vegetables, olive oil, and legumes [20]. Several epidemiological studies have suggested that adherence to Western dietary patterns is associated with an increased risk of breast cancer and, inversely, that a Mediterranean diet may favorably reduce the risk of breast cancer [21, 22].

The 32.89% total variance explained compares favorably to prior Iranian studies (range 25.8%–31.2%) [9, 11] and international studies (median 29.4%) [8, 22], while capturing culturally unique aspects of southwestern Iranian diets. While the explained variance suffices to identify principal dietary axes, future studies could expand food lists to capture regional specialties, employ longitudinal designs to assess pattern stability, and integrate biomarker validation to complement FFQ data.

The recent meta‐analysis of 15 cohort and 34 case–control studies in Asian populations found that a healthy diet with a high intake of vegetables, fruit, and soy significantly reduced the risk of breast cancer. In contrast, an unhealthy diet high in meat and animal products was associated with an increased risk of breast cancer [8]. The association between major dietary patterns and the risk of breast cancer in Iranian women has been investigated in a few studies. In line with our findings, a hospital‐based case–control study showed that following a healthy dietary pattern with a high intake of whole grains, legumes, seeds, fruits, vegetables, olive oil, and liquid olive oil, as well as avoiding salt intake, fish, and seafood, was not associated with an increased risk of breast cancer. However, an unhealthy dietary pattern with a high intake of processed meat, sweets, solid oils, soft drinks, fried and boiled potatoes, mayonnaise, and salt was associated with a significantly increased risk of breast cancer [11]. Karimi et al. [9] reported in another hospital‐based case–control study that a healthy dietary pattern, defined as high consumption of whole grains, fruits, vegetables, legumes, soy, spices, low‐fat dairy products, fish, poultry, organ meats, pickles, olive oil, and vegetable oils, was associated with a 75% reduced risk of breast cancer. On the other hand, a high intake of refined cereals, nuts, soft drinks, industrial juice, tea and coffee, sugar, sweets and desserts, hydrogenated fats, French fries, and potato chips, red and processed meats, and salt was associated with a significantly increased risk of breast cancer. Similarly, the protective effect of a healthy dietary pattern on breast cancer and the strong positive association between an unhealthy dietary pattern and the risk of breast cancer were found in another case–control study among Iranian women [23, 24, 25].

While previous Iranian studies pooled pre‐ and postmenopausal women [23, 24, 25], we identified a 4.22‐fold increased risk linked to Western diets exclusively in premenopausal women, a novel finding suggesting hormonal interactions specific to this subgroup. The etiology and risk factors for breast cancer differ according to menopausal status, as estrogens have been proposed to be important in the development of breast cancer, and the source and metabolism of estrogens differ between premenopausal and postmenopausal women [26]. Therefore, we further performed the stratified analysis to examine the association between dietary patterns and breast cancer by menopausal status. It is interesting to note that the Western dietary pattern was associated with a 4.22‐fold increased risk of breast cancer in pre‐menopausal women, but not in postmenopausal women. The lack of significant links between dietary patterns and breast cancer risk in postmenopausal women may stem from biological, cultural, and methodological factors. Biologically, postmenopausal breast cancer is more influenced by adiposity and metabolic syndrome rather than diet‐driven hormonal pathways, which are more relevant in premenopausal women [27]. Culturally, older Iranian women often maintain traditional diets, resulting in reduced dietary variability, whereas younger urban women tend to adopt more Westernized eating habits [28]. In addition, the limited sample size (*n* = 44 postmenopausal cases), residual confounding (e.g., obesity), and the FFQ's focus on recent (rather than lifelong) intake may obscure the associations.

In the current study, the Western dietary pattern included dietary sources of simple carbohydrates and saturated fatty acids, such as refined grains, sweets, sugary drinks, and red and processed meats. The study also identified traditional dietary patterns that include salty foods, tea and coffee, high‐fat dairy products, snacks, white meat (such as poultry, fish, and fish products), eggs, solid oils, and whole grains, which are part of the Iranian dietary culture. To the best of our knowledge, no study has investigated the association between breast cancer and Western and traditional dietary patterns in Iranian women. Lu et al. [29] assessed the association of healthy, Western, and traditional Chinese dietary patterns in a hospital‐based case–control study. They found that the traditional Chinese diet may be favorably associated with breast cancer risk in Chinese women. According to Iran's food culture, the composition of different types of food can interact to enhance or diminish the health effects of food. Further studies are therefore needed to confirm the association between traditional Iranian dietary patterns and breast cancer risk.

Overall, the study identified three major dietary patterns, each characterized by distinct food groups and serving sizes. The Healthy Dietary Pattern emphasized plant‐based foods, including 2–3 servings/day of vegetables (e.g., leafy greens, tomatoes), 2–3 servings/day of fruits (e.g., apples, citrus), and 1–2 servings/day of legumes and nuts (e.g., lentils, almonds), alongside 1–2 servings/day of liquid oils (e.g., olive oil) and 1–2 servings/day of low‐fat dairy. These foods align with established anti‐inflammatory and antioxidant properties, which may contribute to reduced breast cancer risk [30]. In contrast, the Western Dietary Pattern was marked by 3–4 servings/day of refined grains (e.g., white bread), 2–3 servings/week of red/organ meats, and frequent consumption of SSBs (1–2 servings/day) and fast food (1–2 servings/week). The observed association with elevated breast cancer risk (particularly in pre‐menopausal women) underscores the detrimental role of ultra‐processed foods and added sugars. The Traditional Dietary Pattern featured 3–4 servings/day of tea/coffee, 2–3 servings/day of salty foods, one serving/day solid oil (e.g., butter), 2–3 servings/week of white meat (poultry and fish), and 1–2 servings/day of high‐fat dairy, reflecting local culinary habits. These quantified servings provide actionable insights, underscoring the protective potential of the Healthy pattern, rich in fiber, antioxidants, and unsaturated fats, while highlighting the risks associated with processed and energy‐dense foods in the Western pattern.

High intakes of fruit, vegetables, and healthy oils characterize a healthy dietary pattern. Vegetables and Fruits are rich in a combination of micronutrients with potential anti‐cancer activities, including antioxidant vitamins such as folate, vitamin C, and vitamin E, as well as phytochemicals like phenolic compounds, flavonoids, and lycopene, along with dietary fiber. Such nutrients can interfere with carcinogenic pathways by influencing the oxidative stress and immune system, modifying the hormonal status, altering the cell membranes in function and structure, and regulating gene expression and cell signaling pathways [30]. In contrast, the presence of oncogenic compounds could be the possible mechanism for the increased breast cancer risk due to following an unhealthy dietary pattern. For example, processed and unprocessed meats cooked at high temperatures contain carcinogens such as heterocyclic amines, polycyclic aromatic hydrocarbons, and *N*‐nitrosamines [31]. Additionally, foods high in sugar and fat increase blood glucose and insulin levels. Insulin can cause cell proliferation and tumor growth because it is known to induce cell division. Insulin also increases free estrogen levels by inhibiting the production of a protein called sex hormone‐binding globulin [32].

Although we assessed for active smoking status, the lack of passive smoking data may represent residual confounding, particularly given the high prevalence of secondhand smoke exposure among non‐smoking women in Iran. Recent studies estimate that 60%–70% of Iranian non‐smoking women are regularly exposed to secondhand smoke at home or work, with demonstrated associations to breast cancer risk [33]. This limitation should be taken into account when interpreting the associations between our dietary pattern and health outcomes.

The strengths of our study included the high participation rate and the use of a validated dietary assessment tool. In addition, by controlling for several confounders, we tried to find an independent association between dietary patterns and breast cancer risk. In addition, to the best of our knowledge, this is the only study to have identified traditional dietary patterns and examined their association with breast cancer in Iranian women. Additionally, this study emphasizes the role of local dietary transitions (e.g., the rise in adherence to Western dietary patterns in urban Ahvaz) as a modifiable risk factor. In addition, all women with breast cancer had histopathological confirmation. Despite the study's strengths, some limitations should be considered when interpreting the results. It is important to remember that dietary data and other demographic information collected through face‐to‐face questionnaires may not accurately reflect actual dietary habits due to recall bias. Measurement error is unavoidable in any study investigating dietary patterns. However, the recruitment of incident patients, the use of hospital controls, and the administration of valid FFQs by skilled nutritionists minimized these issues in a hospital setting. In addition, while our control group was selected from hospital patients, the strict inclusion criteria suggest they reasonably represent local dietary patterns. However, we acknowledge that population‐based controls might better capture absolute dietary norms; however, such sampling was impractical given the study constraints. Future studies could benefit from mixed hospital‐community control designs. We recognize that while active smoking rates are low among Iranian women, passive smoking exposure is prevalent every day in this population. Our study did not collect specific data on secondhand smoke exposure, which represents an essential limitation given its established association with breast cancer risk in Iranian populations. Future studies in this region should incorporate a detailed assessment of both household and occupational passive smoking exposure. A significant limitation of our study is that it did not exclusively include newly diagnosed cases. While we adjusted for duration of diagnosis and treatment modalities, dietary patterns may have been modified post‐diagnosis, particularly among women within 1 year of diagnosis who might have altered their nutritional intake due to therapy‐related side effects or behavioral changes. Furthermore, our study is limited by the imprecise results obtained due to the small sample size.

## 5 Conclusion

In conclusion, a Western dietary pattern characterized by refined grains, red and organ meats, SSBs, sweets and desserts, and fast food is associated with breast cancer in premenopausal women in Iran. In contrast, a healthy dietary pattern and a traditional dietary pattern do not appear to be mutually associated in this population. However, the relationship between dietary patterns and breast cancer is complex, and there is a need for more studies with a prospective design and on a large scale. Still, this study provides foundational data for future interventions tailored to ethnic subgroups in Southwest Asia.

## Author Contributions

F.B. and H.R. conceived and designed the study. M.H.N. and P.J. collected the data. F.B. and M.R.Z. analyzed data or performed statistical analyses. F.B. and A.V. wrote the manuscript; S.J. and S.R. critically revised the manuscript for important intellectual content. F.B. had primary responsibility. All authors read and approved the final manuscript.

## Ethics Statement

This study was conducted in accordance with the guidelines outlined in the Declaration of Helsinki, and all procedures involving human subjects were approved by the Ethics Committee of the Ministry of Health and Medical Education of Iran and the Ahvaz Jundishapur University of Medical Sciences (approval number: IR.AJUMS.REC.1402.248). Written informed consent was obtained from all subjects.

## Consent

The authors have nothing to report.

## Conflicts of Interest

The authors declare no conflicts of interest.

## Data Availability

The data that support the findings of this study are available on request from the corresponding author. The data are not publicly available due to privacy or ethical restrictions.
